# Effects of different doses of dexmedetomidine combined with sufentanil in intravenous controlled analgesia after Salter osteotomy in children

**DOI:** 10.3389/fped.2024.1361330

**Published:** 2024-06-19

**Authors:** Zhiwei He, Huanhuan Ni, Wei Wang

**Affiliations:** ^1^ Department of Anaesthesiology, Children’s Hospital of Fudan University, Shanghai, China; ^2^ Department of Anaesthesiology, Children’s Hospital of Nanjing Medical University, Nanjing, China

**Keywords:** dexmedetomidine, postoperative analgesia, sufentanil, developmental hip dislocation, Salter osteotomy

## Abstract

**Background:**

This study aimed to investigate the effect of different doses of dexmedetomidine combined with sufentanil on postoperative analgesia in developmental hip dislocation in children after Salter osteotomy.

**Methods:**

The clinical data of 98 children with developmental hip dislocation, who underwent Salter osteotomy in our center between January 2020 and February 2023, were selected. The children were randomly divided into four groups based on the application of patient-controlled intravenous analgesia (sufentanil + granisetron ± dexmedetomidine). All children received 1 µg/kg/day of sufentanil and 3 mg of granisetron. Group A did not receive dexmedetomidine, and Groups B, C, and D received 0.5, 0.75, and 1.0 µg/kg/day of dexmedetomidine, respectively. The pain indicators and immune factor levels of children in each group were compared.

**Results:**

The heart rate (HR) and respiratory rate (RR) 2 h after operation in Groups C and D were significantly lower than those in Groups A and B (*P *< 0.05). The pain scores decreased over time after treatment in all groups. When compared at the same time point, children in Group D had the lowest pain scores, which were significantly lower than the other three groups (*P *< 0.05). The total consumption of sufentanil in Groups C and D was significantly lower than that in Group A (*P *< 0.05). On the first day after surgery, the children in Group D had lower levels of serum adrenocorticotropic hormone, interleukin-6, and corticosterone than those in Group A (*P *< 0.05).

**Conclusion:**

Administration of 1.0 µg/kg/day of dexmedetomidine combined with sufentanil in intravenous controlled analgesia after Salter osteotomy for developmental hip dislocation in children has a better analgesic effect, less consumption of sufentanil, and low incidence of opioid adverse reactions.

## 1 Introduction

Salter osteotomy is an effective operation for developmental hip dislocation in children. Postoperative pain caused by surgical trauma and drainage tube stimulation brings great pain to children and delays their recovery (1). Postoperative pain is one of the main aspects of postoperative discomfort, which increases the incidence of postoperative complications, prolongs the hospitalization time, or evolves into chronic pain and brings serious psychological and physiological harm to children (2). Perfect postoperative analgesia can inhibit the excessive excitation of the sympathetic nerve, can reduce oxygen consumption, reduce the catecholamine concentration in blood, can reduce the occurrence of cardiovascular and cerebrovascular adverse events, and is conducive to the recovery of children after surgery (3). Dexmedetomidine hydrochloride is a novel selective agonist of the A2 adrenoceptor, which has the effects of sedation, analgesia, anti-anxiety, hypnosis, amnesia, anti-sympathetic, anti-stress, and myocardial and cerebral protection (4, 5). Studies have shown that dexmedetomidine combined with morphine for postoperative analgesia can yield a good analgesic effect and that the incidence of nausea and vomiting is low (6). At present, sufentanil is commonly used as an intravenous opioid analgesic in China. However, there are few studies on the safety and effectiveness of dexmedetomidine combined with sufentanil for patient-controlled intravenous analgesia in the literature search. Clinical research in this area is needed to obtain relevant experience and basis. Studies have shown that dexmedetomidine can further optimize the analgesic effect of sufentanil (7). The purpose of this study was to investigate the effect of dexmedetomidine combined with sufentanil on postoperative analgesia in children with developmental hip dislocation after Salter osteotomy by observing the safety, effectiveness, patient satisfaction, and saving effect of sufentanil and dexmedetomidine in postoperative analgesia to provide a reference for the clinical application of postoperative analgesia in children.

## 2 Data and method

### 2.1 Clinical data

**Figure 1:**
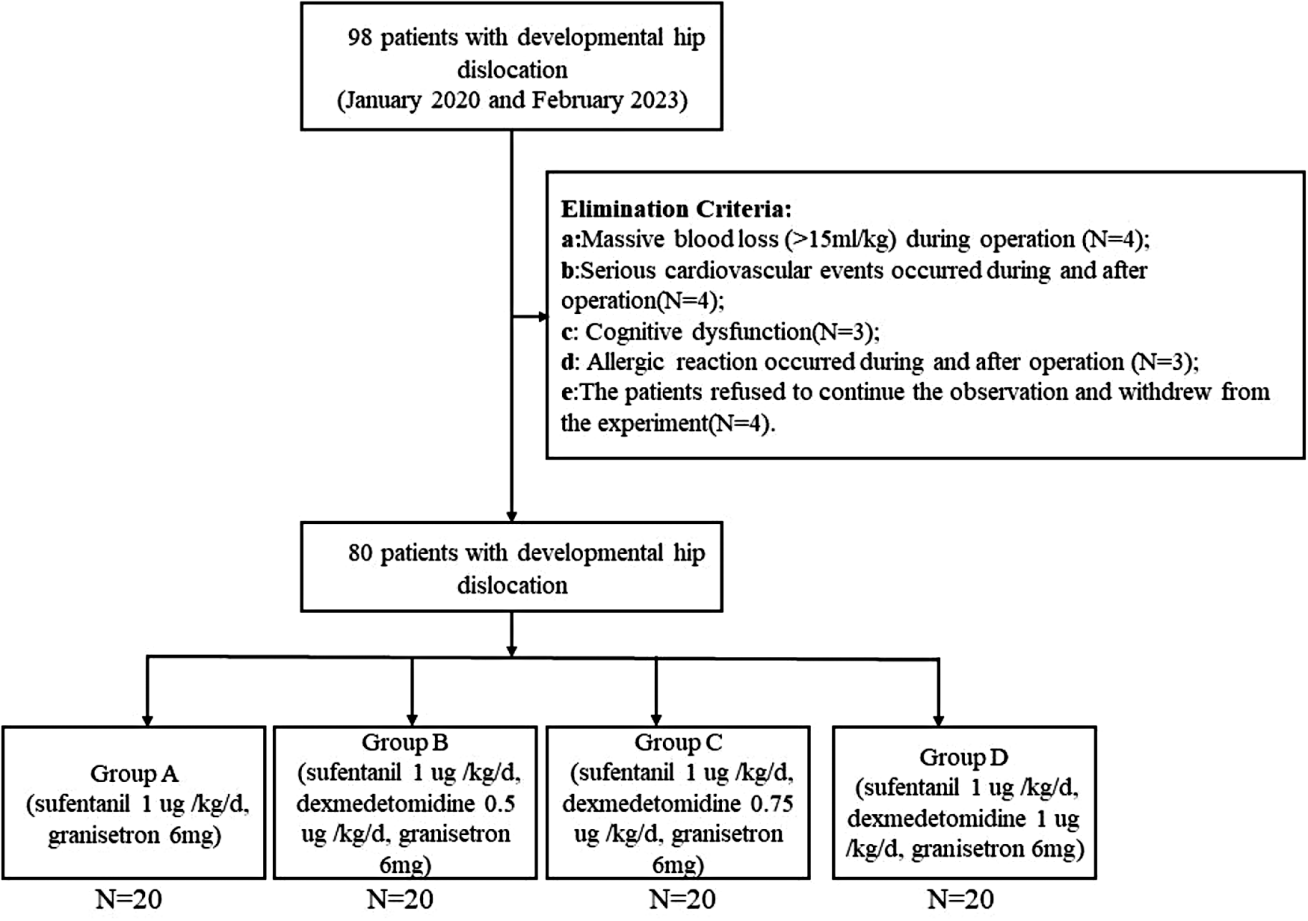
The flowchart.

The clinical data of 98 children with developmental hip dislocation who underwent Salter osteotomy in our center between January 2020 and February 2023 were selected. The children were divided into four groups according to the application of controlled analgesic drugs in a 1:1 ratio by employing a random number generator in Microsoft Excel (flowchart shown in Figure 1). The Ethics Committee of the Children's Hospital of Fudan University approved this study (2020KY-122).

### 2.2 Inclusion criteria

After obtaining approval from the hospital's ethics committee and consent from patients and families, 5- to 8-year-old patients under the American Society of Anesthesiologists (ASA) I to II were selected, and general anesthesia was administered.

### 2.3 Exclusion criteria

The exclusion criteria were as follows: (1) history of bradycardia, atrioventricular block, or taking β-receptor blockers (metoprolol, atenolol, etc.); (2) serious cardiovascular diseases; (3) abnormal liver and kidney function; (4) hypoproteinemia; (5) history of allergy to the drugs used in this test; (6) history of drug addiction (including opioids, sedative-hypnotic drugs, and anti-anxiety drugs); (7) taking sedatives or antidepressants; and (8) diseases of the central nervous system.

### 2.4 Elimination criteria

The elimination criteria were as follows: (1) massive blood loss (>15 ml/kg) during operation; (2) serious cardiovascular events that occurred during and after operation; (3) cognitive dysfunction; and (4) allergic reaction that occurred during and after operation.

## 3 Research methodology

### 3.1 Anesthetic methods

(1) All children had no preoperative medication.
(2) Electrocardiogram (ECG), blood pressure (BP), and SpO2 were monitored routinely after entering the room. Oxygen was given by face mask with a flow rate of 3 L/min after the upper limb venous access was established. The induction started 5 min later.
(3) Anesthesia induction: 0.04 mg/kg of midazolam and 0.4 μg/kg of sufentanil were injected intravenously, and then 1.5–2 mg/kg of propofol was injected slowly. When the patient's observer's assessment alert/sedation score was W2 (no response to name calling, only response to tapping or pushing), 0.9 mg/kg of rocuronium was immediately injected intravenously. Tracheal intubation was performed 3 min after manual respiration.
(4) Anesthesia maintenance: 4–10 mg/kg/h of propofol was used for total intravenous maintenance during the operation; 0.1–0.2 µg/kg/min of remifentanil and besylate 2 µg/kg/min of cisatracurium were then administered. The heart rate (HR), BP, SpO2, and PETCO2 were monitored during the operation, and BIS was maintained at 40–60. All children received 3 mg of granisetron and 0.2 µg/kg of sufentanil intravenously 30 min before the end of the surgery, and cisatracurium besylate was stopped. The infusion of propofol and remifentanil was also stopped.
(5) Indication of extubation: Can open eyes and raise head according to oral command, tidal volume of over 5 ml/kg, respiratory rate (RR) of 16–25 times/min, and stable circulation.
(6) Postoperative analgesia: The children in each group received an intravenous analgesia pump within 3 days after operation. All children received 1 µg/kg/day of sufentanil and 6 mg of granisetron. Group A did not receive dexmedetomidine, and Groups B, C, and D received 0.5, 0.75, and 1.0 µg/kg/day of dexmedetomidine, respectively, which was made into 150 ml with a 0.9% sodium chloride solution. The first dose of each group was 2 ml, the background dose was 2 ml/h, the patient-controlled analgesia (PCA) dose was 2 ml/time, the lockout time was 15 min, and the limit dose was 13 ml/h. When PCA does not relieve pain, analgesics, such as acetaminophen and NSAID, can be used remedially.

### 3.2 Observational indicators

(1) General information including age, sex, height, weight, past medical history, complications, medication history, and drug allergy history.
(2) Visual analog scale (VAS): the degree of pain was evaluated by VAS 30 min and 1, 2, 4, 12, 24, and 48 h after operation (8). A 10-cm-long line with a scale of 0–10 is used to represent the degree of pain, where 0 is completely painless and 10 is very intense pain and unbearable. The patient marks the position on the line that is most consistent with the pain degree and measures the scale of the corresponding position with a ruler, that is, the pain score of the patient. VAS = 0 means the patient does not feel pain at all; VAS = 1–3 means the patient feels slight pain, but tolerable; VAS = 4–6 means the patient feels moderate pain causing disturbed sleep, but tolerable; VAS = 7–10 means the patient feels severe and unbearable pain causing disturbed sleep.
(3) The total dose of sufentanil, the total number of PCA, and the effective number of PCA at 48 h.
(4) Mean arterial pressure (MAP) is associated with increased levels of HR, RR, and SpO2.
(5) Children's general satisfaction score (0–100) (9) and Ramsay's sedation score (10). Grade I, anxiety and/or dysphoria; Level II, quiet cooperation, accurate orientation; Class III, responsive only to instructions; Grade IV, the patient is asleep, gently tapping the glabella or loudly calling when the reaction is agile; Class V, the patient is drowsy and slow to respond to a tap on the brow or a loud call; Grade VI, the patient is asleep and unresponsive to various stimuli. The ideal sedative state is Levels II–V
(6) Adverse events, such as VAS >4, nausea and vomiting, respiratory depression, and cardiovascular events were recorded.
  • Nausea scores (11): 0 point, no nausea; 1 point, not feeling nausea when resting and lying quietly, with slight nausea when moving; 2 points, having intermittent nausea at rest; and 3 points, experiencing persistent nausea at rest and increased nausea during activity.
  • Vomiting score: 0 point, no vomiting; 1 point, mild vomiting, about 1–2 times in 24 h; 2 points, moderate vomiting, 3–5 times in 24 h; and 3 points, severe vomiting, more than five times in 24 h.
  • Treatment of adverse events (12): fentanyl citrate 50 µg was given intravenously when the VAS was >4 and unsatisfactory. If the VAS was >4 and still unsatisfactory, the patient was withdrawn from the trial, and the analgesic protocol was changed. When the HR was <50 beats/min, atropine was injected intravenously at 3 mg each time and repeated if necessary. Vasoactive drugs such as esmolol, percipience, ephedrine, and phenylephrine should be used when the BP is higher or lower than 30% of the basic value. When nausea and vomiting occurred, children with a nausea score of >2 or a vomiting score of >1 received 3 mg of granisetron intravenously. When severe respiratory depression occurred (RR, <8/min; duration, >10 min), naloxone was given intravenously at 0.2 mg and repeated if necessary.
(7) Enzyme-linked immunosorbent assay (ELISA): fasting venous blood was obtained from each child in the morning on the day before the operation and the first and second days after operation. The serum levels of the adrenocorticotropic hormone (ACTH), interleukin-6 (IL-6), and cortisol (COR) were detected using ELISA kits (R&D Systems, USA).

### 3.3 Statistical analysis

SPSS 17.0 software was used for data analysis. Data were presented as mean ± standard deviation, ($\bar{x}$±s). One-way ANOVA with a repeated-measures design was used to compare the two groups. Enumeration data were compared using the $\chi^{2}$ test, and rank data were compared using the rank-sum test. A *P*-value of <0.05 was considered statistically significant.

## 4 Result

### 4.1 Comparison of the general information on children

There were no significant differences in age, weight, operation time and intraoperative opioid dosage among the four groups (*P *> 0.05) (Table 1).

**Table 1:** Comparison of the general conditions and operation conditions of the four groups ($\bar{x}$ ± s).

Group	Age (years)	Weight (kg)	Height (cm)	Operation time (min)	Remifentanil (this/kg)	Infusion volume (ml)
Group A	6.4 ± 0.7	23.3 ± 4.8	121.3 ± 6.1	82.5 ± 15.6	15.8 ± 4.3	1,200 ± 245
Group B	6.7 ± 1.0	25.3 ± 3.6	120.5 ± 7.5	85.3 ± 13.4	15.0 ± 4.7	1,155 ± 295
Group C	6.5 ± 0.9	23.6 ± 4.0	123.2 ± 5.5	81.6 ± 16.0	16.4 ± 5.0	1,252 ± 336
Group D	6.5 ± 0.8	24.9 ± 3.5	122.5 ± 7.3	83.8 ± 15.1	15.1 ± 4.6	1,195 ± 278

### 4.2 Comparison of vital signs (systolic blood pressure, diastolic blood pressure, HR, and respiration rate) at 30 min and 1, 2, 4, 12, 24, and 48 h after operation

No significant difference was observed in the systolic blood pressure (SBP) and diastolic blood pressure (DBP) among the four groups (*P *> 0.05) (see Table 2). The HR and RR at 2 h after operation in Groups C and D were significantly lower than those in Groups A and B (*P *< 0.05).

**Table 2:** Comparison of the vital signs of children in the four groups at different postoperative time points ($\bar{x}$ ± s).

Group		30 min	1 h	2 h	4 h	12 h	24 h	48 h
Group A	HR (times/min)	108.4 ± 7.3	104.4 ± 12.	97.7 ± 13.4	87.6 ± 15.0	78.2 ± 13.5	77.3 ± l4.5	76.4 ± 12.9
	SBP (mmHg)	117.4 ± 18.5	111.3 ± 22.3	114.3 ± 25.1	l13.7 ± 30.0	112.4 ± 23.2	116.5 ± 18.7	114.1 ± 20.5
	DBP (mmHg)	83.7 ± 8.4	75.4 ± 11.4	73.4 ± 9.6	78.9 ± 14.6	77.7 ± 10.3	75.8 ± 8.2	76.4 ± 7.3
	RR (times/min)	27.3 ± 5.5	26.6 ± 6.1	26.3 ± 3.5	26.9 ± 6.4	25.1 ± 3.6	24.8 ± 4.7	24.4 ± 4.6
Group B	HR (times/min)	107.6 ± 8.6	106.3 ± 8.4	88.5 ± 9.3	84.7 ± 7.6	79.5 ± 6.7	74.3 ± 7.7	75.4 ± 8.9
	SBP (mmHg)	116.4 ± 14.8	114 ± 21.8	116.2 ± 18.1	112 ± 19.0	115 ± 19.7	117 ± 17.8	113 ± 18.5
	DBP (mmHg)	81.5 ± 8.5	78.5 ± 9.5	76.5 ± 8.3	78.5 ± 8.7	75.6 ± 7.4	77.5 ± 6.5	76.0 ± 4.9
	RR (times/min)	27.1 ± 5.4	25.5 ± 5.5	23.6 ± 4.6	22.3 ± 5.1	21.8 ± 5.4	21.1 ± 3.5	20.3 ± 3.0
Group C	HR (times/min)	106.9 ± 9.5	97.4 ± 7.3	83.4 ± 8.6**	74.7 ± 5.9	72.6 ± 6.8	72.8 ± 8.5	74.2 ± 7.7
	SBP (mmHg)	119.2 ± 12.3	117.6 ± 24.6	118.4 ± 19.3	120.3 ± 16.9	116.8 ± 22.2	120.2 ± 21.7	119.8 ± 22.3
	DBP (mmHg)	82.3 ± 6.2	71.5 ± 7.6	73.4 ± 8.8	74.9 ± 7.8	73.8 ± 6.5	74.8 ± 5.2	73.1 ± 6.4
	RR (times/min)	27.6 ± 3.6	24.2 ± 4.4	20.8 ± 4.5**	20.5 ± 5.1	20.1 ± 4.2	19.4 ± 4.0	19.3 ± 3.5
Group D	HR (times/min)	107.4 ± 8.5	96.6 ± 7.4	85.6 ± 6.8**	73.7 ± 7.5	74.7 ± 5.9	72.8 ± 7.7	74.8 ± 5.4
	SBP (mmHg)	119.4 ± 20.5	121.5 ± 22.6	117.8 ± 18.7	118.6 ± 19.8	119.3 ± 20.3	113.4 ± 18.4	117.8 ± 21.5
	DBP (mmHg)	83.3 ± 5.6	76.5 ± 8.6	73.4 ± 9.6	79.5 ± 8.4	72.5 ± 7.9	77.2 ± 6.2	78.6 ± 4.3
	RR (times/min)	26.3 ± 4.6	23.5 ± 5.4	20.3 ± 4.5**	19.8 ± 5.8	19.5 ± 4.0	18.5 ± 7.0	18.4 ± 4.4

Heart rate, HR; systolic pressure, SBP; diastolic pressure, DBR; respiration rate, RR.

*Compared to Group A, *P *< 0.05.

** Compared to Group B, *P *< 0.05.

### 4.3 Comparison of postoperative pain VAS scores of children

The pain scores decreased over time after treatment in all groups. When comparing pain scores at the same time point, children in Group D had the lowest scores, which were significantly lower than those of the other three groups (*P *< 0.05). This was followed by Group C, whose scores were significantly lower than Groups A and B (*P *< 0.05) (Table 3).

**Table 3:** Postoperative pain in the four groups ($\bar{x}$ ± s).

Group	1 h	2 h	4 h	12 h	24 h	48 h
Group A	2.6 ± 0.4	3.2 ± 0.6	3.4 ± 0.6	3.1 ± 0.7	2.3 ± 0.6	1.9 ± 0.8
Group B	2.3 ± 0.5*	2.7 ± 0.7*	2.7 ± 0.7	2.6 ± 0.5	2.2 ± 0.8	1.8 ± 0.7
Group C	2.4 ± 0.3**	2.1 ± 0.4**	2.2 ± 0.4**	1.7 ± 0.4**	1.9 ± 0.5**	1.5 ± 0.6**
Group D	1.7 ± 0.7***	1.6 ± 0.6***	1.5 ± 0.6***	1.4 ± 0.5***	1.3 ± 0.3***	1.1 ± 0.3***

* *P *< 0.05 compared to Group A.

** *P *< 0.05 compared to Group B.

*** *P *< 0.05 compared to Group C.

### 4.4 Comparison of effective pressing times of PCA in children

The pressing times of children in Groups B, C, and D were significantly less than those in Group A (*P *< 0.05). The total consumption of sufentanil in Groups C and D was significantly lower than that in Group A (*P *< 0.05) (Table 4).

**Table 4:** Comparison of PCA use in children in the four groups ($\bar{x}$ ± s).

Group	48 h compressions	48 h total sufentanil (gg/kg)
Group A	13.7 ± 3.6	2.53 ± 0.14
Group B	8.4 ± 2.6*	2.27 ± 0.11
Group C	2.4 ± 0.4*	2.08 ± 0.02*
Group D	0.3 ± 0.1*	1.96 ± 0.01*

* *P *< 0.05 compared to Group A.

### 4.5 Comparison of Ramsay sedation scores

**Figure 2:**
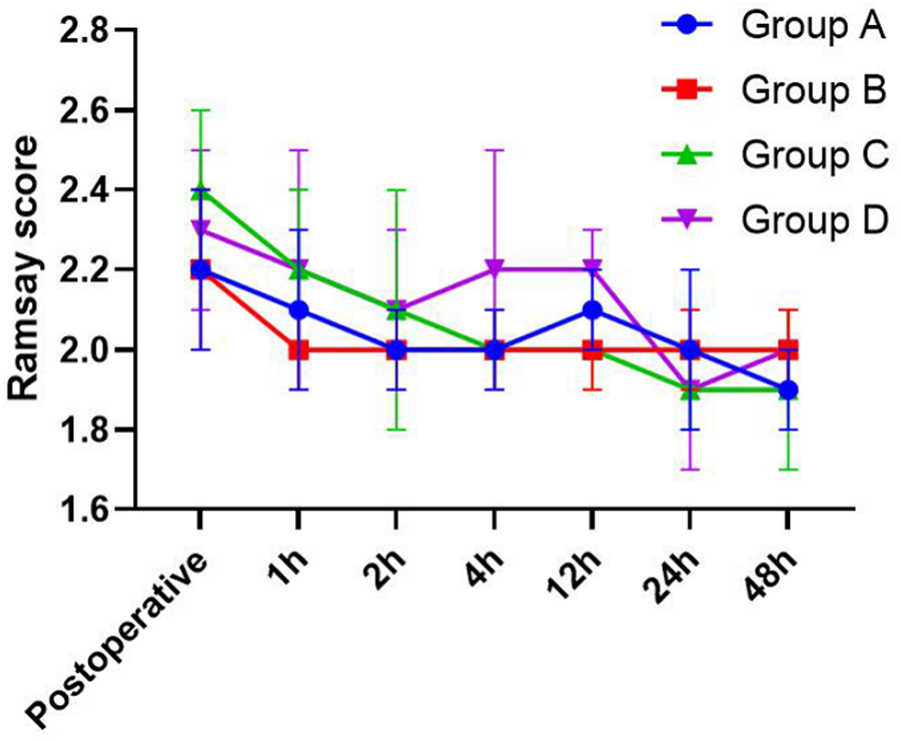
Ramsay sedation scores at each time point after operation.

There was no significant difference in the Ramsay sedation scores among Groups B, C, D, and group A at each time point (Figure 2).

### 4.6 Comparison of adverse effects

Nausea and vomit were observed in children in Groups A and B (Table 5). No adverse effects were observed in children in Groups C and D.

**Table 5:** Comparison of incidence of adverse reactions.

Group	Number	Nausea (%)	Vomit (%)	Respiratory (%)	Chill (%)
Group A	20	25	10	0	10
Group B	20	15	5	0	0
Group C	20	0*	0	0	0
Group D	20	0*	0	0	0
Totally	80	10	3.75	0	2.5

* *P *< 0.05 compared to Group A.

### 4.7 Comparison of ACTH, IL-6, and COR levels

**Figure 3:**
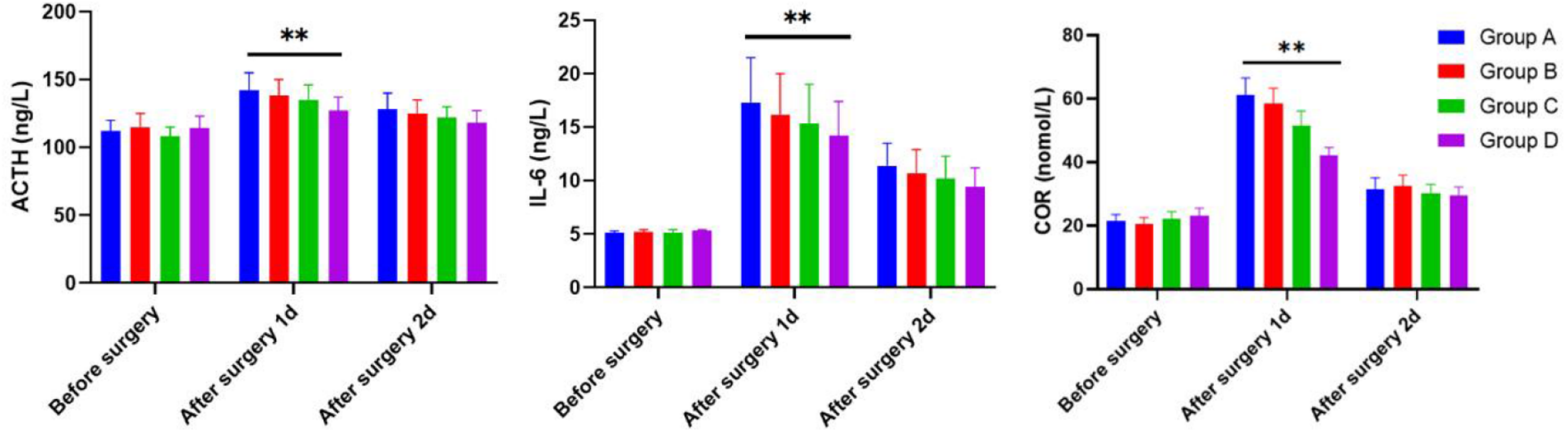
The serum levels of ACTH, IL-6, and COR in children 1 day before surgery and 1 and 2 days after operation of the four groups as determined by the ELISA methods.

The ACTH, IL-6, and COR serum levels of children were determined by the ELISA methods 1 day before surgery and 1 and 2 days after surgery across Groups A, B, C, and D. As shown in Figure 3, all groups exhibited no significant difference concerning ACTH, IL-6, and COR serum levels of children on the first day before surgery (*P *> 0.05). On the first day after surgery, Group D had lower serum levels of ACTH, IL-6, and COR than those in Group A. On the second day after surgery, no significant difference was noted in the serum levels of ACTH, IL-6, and COR in children across the four groups (*P *> 0.05).

### 4.8 Comparison of the overall satisfaction of children

The number of children who reported high satisfaction increased gradually in Groups A–D, and the satisfaction rate increased gradually (Table 6, *P *< 0.05).

**Table 6:** Comparison of children's overall satisfaction.

Group	Number	Very satisfied	Satisfied	Dissatisfied	Rate of overall satisfaction
Group A	20	3	14	3	85%
Group B	20	10	8	2	90%
Group C	20	19*	1*	0*	100%
Group D	20	20*	0*	0*	100%
Totally	80	52	23	5	93.75%

* *P *< 0.05 compared to Group A.

## 5 Discussion

Effective postoperative analgesia depends on the reasonable compatibility of analgesic drugs. Currently, clinics have used many kinds of postoperative analgesics, which are mainly divided into two categories: opioids and non-opioids (13–15). Common opioids include morphine and fentanyl, whereas non-opioid drugs include non-steroidal anti-inflammatory drugs and alpha-2 adrenoceptor agonists (16–19). Opioids are the most potent analgesic drugs discovered thus far, and their analgesic effects increase with the dose. The analgesic mechanism of opioids is multilevel. They can bind to pain opioid receptors in peripheral nerves and sensory neurons in the glial layer of the spinal dorsal horn, brain, and brain stem, inhibit the release of Substance P (SP) at the spinal level, and exert descending pain inhibition at the supraspinal level (20, 21). Large doses of opioids can achieve effective analgesic effects, but following the increase of its side effects, such as nausea, vomiting, constipation, lethargy, and respiratory depression. Thus, how to compound the use of opioid and non-opioid drugs, maximize their synergistic analgesic effect, and reduce the dose of opioid drugs to reduce their side effects are the current research focus and difficulty (22, 23). Dexmedetomidine, the dextranomer of medetomidine, is a new type of highly selective alpha-2 adrenoceptor agonist. Clinical studies have confirmed that dexmedetomidine as an anesthetic adjuvant can increase the role of anesthetics and reduce the dosage of anesthetics and has the characteristics of light respiratory depression and small stress response. Dexmedetomidine significantly enhances the role of opioids and reduces their dosage during surgery (24). The results of this experiment also showed that the use of dexmedetomidine (0.75 μg/kg/day, 1 μg/kg/day) combined with sufentanil for postoperative analgesia led to a lower postoperative pain VAS score than that of the simple sufentanil group. The analgesic effect is more effective.

Surgical trauma promotes the release of various inflammatory mediators from the tissue, leading to increased pain effects. SP is a key factor in pain signal transduction, which plays a signal transduction role in nerve conduction (25). Dexmedetomidine has an analgesic effect by inhibiting the release of SP and other nociceptive substances from the presynaptic membrane of the descending medulla–spinal NE pathway. Opioids also inhibit the decrease of cAMP concentration by inhibiting SP and acting on G protein to achieve an analgesic effect (26). Therefore, dexmedetomidine combined with opioids can significantly increase the analgesic effect and play a synergistic role. In addition, remifentanil, an ultrashort-acting opioid, was used for maintenance anesthesia in this study, which may cause acute opioid tolerance or hypersensitivity after surgery (27). This may be related to the upregulation of the cAMP pathway and the activation of the NMDA receptor, which leads to central sensitization. Dexmedetomidine has been reported to inhibit the release of epinephrine and make the release of epinephrine in the spinal intermediate neurons.

Dexmedetomidine selectively activates the postsynaptic a2A/D receptor in the nucleus tractus solitarius (NTS) and inhibits the discharge of sympathetic neurons in the anterior horn of the spinal cord. The activation of the presynaptic a2A/D receptor of the sympathetic nerve terminals inhibits the release of norepinephrine and reduces the concentration of plasma catecholamine, thus maintaining the stability of hemodynamics. This circulatory stabilizing effect is now well recognized. In this study, the pain of children was obvious within 24 h after operation, and catecholamines were released in large quantities, which caused fluctuations in HR and BP (0.75 μg/kg/day, l μg/kg/day). A combination of sufentanil and dexmedetomidine can reduce VAS scores. Dexmedetomidine can activate the postsynaptic alpha-2 adrenoceptor and presynaptic alpha-2 adrenoceptor of the sympathetic nerve, reduce sympathetic tone, and significantly reduce the release of catecholamine *in vivo* by 30%–32%; hence, the combination group has an advantage in cardiovascular stability. The fluctuations of HR, SBP, and DBP in the two-drug combination groups were smaller than those in the sufentanil-alone group. After 48 h, the pain was relieved, and the tolerance of the children was enhanced. Dexmedetomidine (0.75 μg/kg/day, 1 μg/kg/day) was used in combination.

Previous studies have shown that dexmedetomidine can reduce the intraoperative consumption of opioids and other anesthetics. Analysis may show that dexmedetomidine and opioids have a synergistic effect, called the thrifty effect. Because large doses of opioids can achieve effective analgesic effects, side effects, such as nausea, vomiting, and respiratory depression, increase. Sufentanil is commonly used as a postoperative analgesic in clinical work. For children with Salter osteotomy, postoperative pain is severe; if only sufentanil is used for analgesia, a larger dose is needed, and the corresponding side effect rate is high. The experimental results also prove that the analgesic effect of dexmedetomidine combined with sufentanil is significantly increased, and the side effects of sufentanil are effectively reduced.

In this study, the number of PCA effective presses of the combination group in 48 h after surgery was significantly less than the simple drug group. When the dose of dexmedetomidine was 0.75 μg/kg/day or 1 μg/kg/day, the total consumption of sufentanil in 48 h was significantly less than that in the sufentanil-alone group. The above results can be used as dexmedetomidine for postoperative analgesia on sufentanil has a parsimonious effect of evidence.

The side effects of high-dose sufentanil for postoperative analgesia included respiratory depression, nausea, vomiting, urinary retention, and pruritus. In this study, the side effects of the sufentanil-alone group were nausea, vomiting, and shivering. The above reactions were significantly reduced in the combination group. The possible mechanisms are as follows: on the one hand, dexmedetomidine may reduce the incidence of nausea and vomiting by sedation or by putting children in a sleep state; on the other hand, dexmedetomidine can improve the analgesic effect, reduce the demand for sufentanil, reduce sufentanil consumption, thereby reducing the stimulation of pain to the vomiting center. This prevents the adverse reactions caused by large doses of opiates. In this study, no patient demonstrated respiratory depression, which fully shows that dexmedetomidine is safe for children after surgery. Postoperative shivering is a kind of stress reaction after anesthesia, which can lead to increased oxygen consumption and HR, thus increasing the cardiopulmonary load of children and interfering with oxygen saturation and BP, affecting the doctor's judgment. The mechanism may be related to hypothermia during and after an operation, recovery of the thermoregulation center during recovery from anesthesia, and postoperative pain. Many studies have shown that dexmedetomidine can reduce postoperative shivering. The mechanism may be related to the inhibition of catecholamine release and the decrease of the shivering threshold. Shivering occurred in 10% of children in the sufentanil group, but not in the dexmedetomidine-plus-sufentanil group (*P* > 0.05). The possible reason is that the number of cases observed in this experiment is still small, and the significance cannot be observed, which needs to be further explored by increasing the sample size.

The overall satisfaction rate of children in the combination group was significantly higher than that in the sufentanil group. With the increase in dexmedetomidine concentration, the number of people who reported high satisfaction increased, indicating that dexmedetomidine combined with sufentanil can play a significant comfort effect, which may be related to its enhanced analgesic effect and reduce postoperative nausea, vomiting, chills, and other concerns.

To sum up, the results of this study show that 1.0 μg/kg/day of dexmedetomidine combined with sufentanil for children with Salter osteotomy yields precise postoperative analgesic effects. This combination reduces sufentanil consumption and lowers the incidence of opioid-related adverse reactions. Moreover, it plays a good role in stabilizing the cardiovascular system and enhancing patient comfort and satisfaction. However, whether higher doses of dexmedetomidine cause related adverse reactions requires further experimental study.

## Data Availability

The raw data supporting the conclusions of this article will be made available by the authors, without undue reservation.
